# Sepsis: emerging pathogens and antimicrobial resistance in Ethiopian referral hospitals

**DOI:** 10.1186/s13756-022-01122-x

**Published:** 2022-06-13

**Authors:** Melese Hailu Legese, Daniel Asrat, Göte Swedberg, Badrul Hasan, Amha Mekasha, Tadesse Getahun, Misganaw Worku, Eminet Tesfaye Shimber, Seid Getahun, Tsedale Ayalew, Birhan Gizachew, Abraham Aseffa, Adane Mihret

**Affiliations:** 1 Department of Medical Laboratory Sciences, College of Health Sciences, Addis Ababa University, Addis Ababa, Ethiopia; 2 Armauer Hansen Research Institute, Addis Ababa, Ethiopia; 3 Department of Medical Biochemistry and Microbiology, Biomedical Centre, Uppsala University, Uppsala, Sweden; 4 Department of Microbiology, Immunology and Parasitology, School of Medicine, College of Health Sciences, Addis Ababa University, Addis Ababa, Ethiopia; 5 Department of Pediatrics and Child Health, College of Health Sciences, Addis Ababa University, Addis Ababa, Ethiopia; 6 Yekatit 12 Hospital Medical College, Addis Ababa, Ethiopia; 7 Department of Gynecology/Obstetrics, College of Medicine and Health Sciences, Hawassa University, Hawassa, Ethiopia; 8 Department of Emergency and Critical Care Medicine, College of Medicine and Health Sciences, Hawassa University, Hawassa, Ethiopia; 9 Department of Internal Medicine, College of Medicine and Health Sciences, Wollo University, Dessie, Ethiopia; 10 Department of Pediatrics and Child Health, College of Medicine and Health Sciences, Wollo University, Dessie, Ethiopia

**Keywords:** Sepsis etiologies, Emerging pathogens, Multidrug resistance, Multicenter study, Ethiopian referral/teaching hospitals, Ethiopia

## Abstract

**Background:**

Sepsis due to multidrug resistant (MDR) bacteria is a growing public health problem mainly in low-income countries.

**Methods:**

A multicenter study was conducted between October 2019 and September 2020 at four hospitals located in central (Tikur Anbessa and Yekatit 12), southern (Hawassa) and northern (Dessie) parts of Ethiopia. A total of 1416 patients clinically investigated for sepsis were enrolled. The number of patients from Tikur Anbessa, Yekatit 12, Dessie and Hawassa hospital was 501, 298, 301 and 316, respectively. At each study site, blood culture was performed from all patients and positive cultures were characterized by their colony characteristics, gram stain and conventional biochemical tests. Each bacterial species was confirmed using Matrix-Assisted Laser Desorption/Ionization Time-of-Flight Mass Spectrometry (MALDI TOF). Antimicrobial resistance pattern of bacteria was determined by disc diffusion. Logistic regression analysis was used to assess associations of dependent and independent variables. A *p*-value < 0.05 was considered as statistically significant. The data was analyzed using SPSS version 25.

**Results:**

Among 1416 blood cultures performed, 40.6% yielded growth. Among these, 27.2%, 0.3% and 13.1%, were positive for pathogenic bacteria, yeast cells and possible contaminants respectively. *Klebsiella pneumoniae* (26.1%), *Klebsiella variicola* (18.1%) and *E. coli* (12.4%) were the most frequent. Most *K. variicola* were detected at Dessie (61%) and Hawassa (36.4%). Almost all *Pantoea dispersa* (95.2%) were isolated at Dessie. Rare isolates (0.5% or 0.2% each) included *Leclercia adecarboxylata*, *Raoultella ornithinolytica*, *Stenotrophomonas maltophilia*, *Achromobacter xylosoxidans, Burkholderia cepacia, Kosakonia cowanii* and *Lelliottia amnigena*. *Enterobacteriaceae* most often showed resistance to ampicillin (96.2%), ceftriaxone (78.3%), cefotaxime (78%), cefuroxime (78%) and ceftazidime (76.4%). MDR frequency of *Enterobacteriaceae* at Hawassa, Tikur Anbessa, Yekatit 12 and Dessie hospital was 95.1%, 93.2%, 87.3% and 67.7%, respectively. Carbapenem resistance was detected in 17.1% of *K. pneumoniae* (n = 111), 27.7% of *E. cloacae* (n = 22) and 58.8% of *Acinetobacter baumannii* (n = 34).

**Conclusion:**

Diverse and emerging gram-negative bacterial etiologies of sepsis were identified. High multidrug resistance frequency was detected. Both on sepsis etiology types and MDR frequencies, substantial variation between hospitals was determined. Strategies to control MDR should be adapted to specific hospitals. Standard bacteriological services capable of monitoring emerging drug-resistant sepsis etiologies are essential for effective antimicrobial stewardship.

## Introduction

Sepsis is a life-threatening condition resulting from a dysregulated immune response to infection that leads to organ dysfunction [1, 2]. In May 2017, the World Health Organization (WHO) recognized sepsis as a major public health problem and called all United Nations (UN) member states to improve sepsis prevention, recognition and management [3]. In 2020, WHO noted that approximately 20% of all-cause global deaths are due to sepsis [4], which affects 49 million people and causes 11 million deaths globally every year [5]. Sepsis disproportionately affects neonates, pregnant women, elderly, patients with severe comorbidities and people living in low-resource settings [2, 6]. Various species of *Klebsiella, Enterobacter, Acinetobacter, and Pseudomonas, E. coli, and S. aureus* are major sepsis etiologies with regional variation [7, 8].

Though sepsis is treatable [9], antimicrobial resistance (AMR) contributes to worsening the consequences of sepsis from longer hospitalization to death [10, 11]. Resistance to cephalosporins, fluoroquinolones, penicillins, aminoglycosides, monobactams, macrolides and carbapenems is spreading globally [7, 8, 10, 12, 13]. In 2015, WHO announced that AMR represents a major threat to human health [14] and in 2019 the UN declared AMR a great threat facing the global community [15]. Findings from around the world showed increasing levels of multidrug resistance, which is worrying for the future [9, 16].

In sub-Saharan countries, there is scarcity of data on sepsis etiologies [17] though the problem is assumed to be very large because of limited health access and high rates of other infectious diseases [4, 6, 12, 17]. Similarly, data on sepsis etiologies and AMR from Ethiopia are scanty and only studies done at single sites, with limited sample sizes and inaccurate bacteria identification, are available [18–22]. A recent multi-country study published by Sands et al. [23] focused on neonatal sepsis etiologies and AMR in Ethiopia. However, this study had limitations, since it included only a single hospital and neonates from Ethiopia. It is crucial to identify bacterial etiologies and determine their AMR patterns among sepsis patients at a larger scale, in order to guide future sepsis prevention efforts. Hence, this study aimed to determine bacterial sepsis etiologies and their AMR patterns at four hospitals, which serve millions of people in the central, southern and northern parts of Ethiopia.

## Methods

### Study design and study sites

A multicenter study was conducted between October 2019 and September 2020 at four selected hospitals in central, southern and northern Ethiopia. University and referral hospitals which had established microbiology laboratories or a link with a nearby government regional microbiology laboratory were selected purposefully. These were Tikur Anbessa Specialized Hospital (TASH) and Yekatit 12 Specialized Hospital Medical College (Y12HMC) in the central region, Hawassa University Comprehensive Specialized Hospital (HUCSH) in the southern region and Dessie Referral Hospital (DRH) in the northern region.

TASH is the teaching hospital of Addis Ababa University located in Addis Ababa, the capital of Ethiopia. The hospital, with more than 800 beds, is the main referral hospital and the oldest hospital in the country, staffed with the most senior specialists. It provides tertiary level referral diagnoses and treatment for approximately 400,000 patients yearly who referred from all over the country.

DRH is one of the largest public hospitals in the northern part of Ethiopia, located in Dessie town, 400 km from Addis Ababa. It is a referral hospital with 560 beds, providing services for the surrounding areas with an estimated population of 5 million and to residents of the neighboring regions.

Y12HMC is located in Addis Ababa and provides health care services to Addis Ababa residents, referral cases from health centers in Addis Ababa and its bordering regions. The college trains medium- and higher-level health professionals. The hospital has over 300 beds and serves for more than 5 million people in its catchment area.

HUCSH is located in Hawassa city in southern Ethiopia, 280 km from Addis Ababa. HUCSH is one of the largest health facilities in the southern part of the country and provides teaching, public health services and research activities. It serves more than 20 million people locally and in the neighboring regions. Currently, the hospital has over 400 beds and provides patient care to 90,200 outpatients, 18,100 hospitalized patients and 1100 emergency cases annually.

While three hospitals had established microbiology laboratories, DRH had not started performing bacteriological culture and antimicrobial susceptibility testing at the time of this study. Therefore, the nearby located Amhara Public Health Institute-Dessie Branch microbiology laboratory was used for blood culture processing and antimicrobial susceptibility testing.

### Patient recruitment and sample size calculation

All patients with suspected cases of sepsis who sought medical service at the study sites were included in the study population. We used the attending physician’s decision to identify eligible patients as sepsis cases. All age groups were included, but patients who had been on antibiotic treatment within the preceding ten days were excluded from the study. A total of 1416 clinically diagnosed cases of sepsis from different wards were enrolled in the study. The sample size was calculated based on a single sample size estimation formula (n = Z^2^ P (1—P) /d^2^) using a proportion of 50% (P = 0.5) [24], due to lack of previous similar multicenter studies. As this was a multicenter study, increasing the sample size was necessary; hence, a precision (d) of 0.03 was used to maximize the sample size and the accuracy of the prevalence estimate. Z stands for Z statistic with the level of confidence of 95%, which is conventional where the Z value is 1.96. With a 10% non-response rate, the total sample size came to 1174 and distributed equally across the four study sites. Keeping the minimum sample size allocated to each study sites, we enrolled a total of 1,416 patients clinically investigated for sepsis to identify sepsis etiologies and characterize AMR patterns of bacterial isolates.

### Data collection

Professional nurses who had experience of sample collection for blood culture and microbiologists who were working in the bacteriology laboratory were recruited as data collectors. Training and clear instructions were given to all data collectors on sociodemographic and clinical data collection, blood sample collection and transportation to bacteriology laboratories and blood culture processing. A single bottle blood culture system was used because introducing three blood culture bottles for a single patient was found to be difficult at the study sites due to several reasons raised from data collectors including manpower and laboratory settings shortage. Blood cultures, bacterial identification and drug susceptibility testing were performed in accordance with a standardized laboratory protocol that was applied in all study sites. The findings of each blood culture were communicated to attending physicians for patient management. All bacterial strains were stored at − 70 °C and transported to the Armauer Hansen Research Institute (AHRI) and Sweden for further characterization.

### Blood sample collection and transportation

Immediately after the patient was identified, an appropriate blood sample volume was collected and a single blood culture bottle processed for each patient. From children who were < 1, 2–5, 6–11 and 12–17 years old, 1 ml, 2 ml, 3–5 and 5–10 ml was collected, respectively, and from adults, 10 ml. A 1:5 and 1:10 blood to broth dilution was made for children and adults, respectively. Blood samples were collected aseptically using 70% alcohol and 2% tincture of iodine and then transferred to blood culture bottles containing brain heart infusion broth (Oxoid Ltd, UK) and transported to a bacteriology laboratory for culturing and identification.

### Blood cultures and bacterial identification

All blood culture bottles were incubated aerobically at 37 °C for seven consecutive days and inspected daily for signs of bacterial growth. Blood samples that grew turbid before the seventh day and blood samples that were non-turbid on the seventh day were sub-cultured on blood agar (Oxoid Ltd, UK) and MacConkey agar (Oxoid Ltd, UK) at 37 °C for 24 h. At each study site, all positive cultures were characterized and identified by their colony characteristics, gram staining and conventional biochemical tests. Triple sugar iron, indole, urea, citrate, lysine decarboxylase, motility and malonate biochemical media were used for identification of gram-negative bacteria. Biochemical tests used to identify gram-positive bacteria at species level were catalase, coagulase and fermentation on mannitol salt agar. Isolations of coagulase negative staphylococci and *Bacillus* species were considered to be possible contaminants, since a single blood culture bottle was used which limits the ability to differentiate between pathogens and contaminants.

### Bacterial strain confirmation using matrix-assisted laser desorption ionization–time of flight mass spectrometry (MALDI-TOF MS)

All bacteria were re-identified and confirmed using MALDI-TOF MS at the Clinical Microbiology Department of Uppsala University Hospital, Uppsala, Sweden, and Karolinska Institute, Stockholm, Sweden. From fresh cultures, a single colony of bacteria was smeared onto a MALDI-TOF plate and the sample was air-dried. Next, 1 µl formic acid was added to each cell, air-dried, and then 1 µl MALDI matrix solution was applied to the cells and air-dried before reading. MALDI-TOF identification was automatically scored by the system software as between 1 and 3 points. All isolates with score 2 and above were accepted and all results below 1.7 and flagged red were rejected. Samples with score 1.7–2 and flagged yellow were re-analyzed.

### Drug susceptibility testing

The susceptibility to antibiotics of bacterial isolates was analyzed using disk diffusion after 16–18 h of incubation at 37 °C. Each zone of inhibition was measured to the nearest millimeter and interpreted as sensitive, intermediate or resistant based on the standardized table supplied by the Clinical and Laboratory Standards Institute [25]. Using a sterile wire loop, 3–5 pure colonies were picked and emulsified in nutrient broth (Oxoid). Standard inocula were adjusted to 0.5 McFarland units and swabbed onto Muller-Hinton agar (Oxoid). Susceptibility of Gram negative isolates was tested against amikacin (30 μg), ampicillin (10 µg), amoxicillin-clavulanic acid (20/10 µg), ampicillin-sulbactam (10/10 µg), aztreonam (30 µg), cefepime (30 μg), cefotaxime (30 µg), ceftriaxone (30 µg), ceftazidime (30 μg), cefuroxime (30 µg), ciprofloxacin (5 μg), chloramphenicol (30 µg), doxycycline (30 µg), gentamicin (10 µg), meropenem (10 µg), piperacillin-tazobactam (100/10 µg), sulfamethoxazole-trimethoprim (1.25/23.75 µg) and tetracycline (30 µg). Gram positive isolates were tested against penicillin (10 units), vancomycin (30 µg), erythromycin (15 µg), cefoxitin (30 µg) and clindamycin (2 µg) [25]. All antibiotics discs were OXOID products (Oxoid Ltd, UK). A bacterium that was simultaneously resistant to three or more antibiotics in different classes was considered MDR.

### Quality assurance and quality control

At each study site, blood samples were collected and transferred to blood culture broth in accordance with standard operating procedures designed to all sites. Immediately after collection, blood culture bottles were transported to the microbiology laboratory for analysis. Each laboratory test was processed in accordance with established protocols and recorded carefully. Standard operating procedures were followed strictly for each laboratory test. *E*. *coli* ATCC 25,922 and/or *Staphylococcus aureus* ATCC 25,923 were used as quality control strains for culture and drug susceptibility testing throughout the study. Each MALDI-TOF run also included quality control strains using *E*. *coli* ATCC 25,922.

### Statistical analysis and interpretation

Descriptive statistics (mean, percentages or frequency and standard deviation) were calculated. Association of possible risk factors with sepsis was assessed using univariate and multivariate logistic regression analysis. A *p*-value < 0.05 was considered as statistically significant. The data were analyzed using SPSS version 25.

## Results

### Blood culture findings

In the present study, a total of 1,416 patients from four different hospitals were investigated for sepsis. The number of patients from TASH was 501, and the numbers from Y12HMC, DRH and HUCSH were 298, 301 and 316, respectively (Table 1). Among all 1416 blood cultures performed, 40.6% yielded growth of 27.2% pathogenic bacteria, 0.3% yeast cells and 13.1% possible contaminants (Table 1). Double bacterial isolates were detected in 2.9% of blood cultures. The highest proportion of positive cultures (38.2%) was detected at DRH, followed by HUCSH (29.1%). Blood cultures performed at TASH and Y12HMC yielded 24.6% and 18.5% bacterial growth, respectively. Contamination rates were 17.1%, 15.2%, 14.6% and 8.6% at Y12HMC, HUCSH, DRH and TASH, respectively. The highest frequency of bacterial pathogens isolated was among neonates < 29 days (39.2%), followed by age groups 1–5 years (24.4%) and ≥ 18 years (18.1%).

**Table 1:** Blood culture findings of patients investigated for sepsis at four different hospitals in Ethiopia

	Sociodemographic data			Blood culture result		
		n (%)	Bacterial growth n (%)	No bacterial growth n (%)	Possibly contaminants n (%)	Yeast cells n (%)
Hospitals	TASH	501 (35.4)	123 (24.6)	335 (66.9)	43 (8.6)	0(0.0)
	Y12HMC	298 (21)	55 (18.5)	191 (64.1)	51 (17.1)	1 (0.3)
	DRH	301 (21.3)	115 (38.2)	142 (47.2)	44 (14.6)	0 (0.0)
	HUCSH	316 (22.3)	92 (29.1)	173 (54.7)	48 (15.2)	3 (0.9)
Gender	Male	783 (55.3)	210 (26.8)	462 (59)	108 (13.8)	3 (0.4)
	Female	633 (44.7)	175 (27.6)	379 (59.9)	78 (12.3)	1 (0.2)
Age category	< 29 days	586 (41.4)	230 (39.2)	258 (44)	94 (16)	4 (0.7)
	≥ 30 days to ≤ 1 year	256 (18.1)	45 (17.6)	182 (71.1)	29 (11.3)	0 (0.0)
	1–5 years	135 (9.5)	33 (24.4)	84 (62.2)	18 (13.3)	0 (0.0)
	5–18 years	158 (11.2)	26 (16.5)	111 (70.3)	21 (13.3)	0 (0.0)
	≥ 18 years	281 (19.8)	51 (18.1)	206 (73.3)	24 (8.5)	0 (0.0)
Ward	EOPD	104 (7.3)	15 (14.4)	79(76)	10 (9.6)	0 (0.0)
	ICU	38 (2.7)	8 (21.1)	28 (73.7)	2 (5.3)	0 (0.0)
	Medical ward	148 (10.5)	24 (16.2)	111 (75)	13 (8.8)	0 (0.0)
	NICU	596 (42.1)	232 (39.9)	265 (45.5)	95 (15.9)	4 (0.7)
	Pediatrics	497 (35.1)	94 (18.9)	342 (68.8)	61 (12.3)	0 (0.0)
	Surgical ward	33 (2.3)	12 (36.4)	16 (48.5)	5 (15.2)	0 (0.0)
Referral patient	Yes	722 (51)	190 (26.3)	444 (61.5)	87 (12)	1 (0.1)
	No	694 (49)	195 (28.1)	397 (57.2)	99 (14.3)	3 (0.4)
Previous admission	Yes	299 (21.1)	69 (23.1)	197 (65.9)	32 (10.7)	1 (0.3)
	No	1117 (78.9)	316 (28.3)	644 (57.7)	154 (13.8)	3 (0.3)
Hospital stay duration	1 week	828 (58.5)	244 (29.5)	464 (56)	117 (14.1)	3 (0.4)
	2 weeks	222 (15.7)	52 (23.4)	141 (63.5)	28 (12.6)	1 (0.5)
	3 weeks	146 (10.3)	35 (24)	97 (66.4)	14 (9.6)	0 (0.0)
	4 weeks and above	220 (15.5)	54 (24.5)	139 (63.2)	27 (12.3)	0 (0.0)
Underlying diseases	Yes	665 (47)	159 (23.9)	433(65.1)	71(10.7)	2 (0.3)
	No	751 (53)	226 (30.1)	408(54.3)	115(15.3)	2 (0.3)
Previous antibiotic treatment before recruitment to the study	Yes	440 (31.1)	121 (27.5)	269(61.1)	48(10.9)	2 (0.5)
	No	976 (68.9)	264 (27)	572(58.6)	138(14.1)	2 (0.2)
Fever	Up to 3 days	687 (64.4)	210 (30.6)	380 (55.3)	95 (13.8)	2 (0.3)
	4–6 days	207 (19.4)	48 (23.2)	144 (69.6)	15 (7.2)	0 (0.0)
	7 days and above	173 (16.2)	31 (17.9)	122 (70.5)	20 (11.6)	00 (0.0)
	No	349 (24.6)	96 (27.5)	195 (55.9)	56 (16.0)	2 (0.6)
	Total	1416 (100)	385 (27.2)	841 (59.4)	186 (13.1)	4 (0.3)
BMI class	Underweight	749 (72.2)	173 (23.1)	472 (63)	102 (13.6)	2 (0.3)
	Normal	233 (22.4)	47 (20.2)	163 (70)	23 (9.9)	0 (0.0)
	Overweight	26 (2.5)	7 (26.9)	18 (69.2)	1 (3.8)	0 (0.0)
	Obese	30 (2.9)	11 (36.7)	17 (56.7)	2 (6.7)	0 (0.0)
	Total for BMI	1038 (100)	238 (22.9)	670 (64.5)	128 (12.3)	2 (0.2)

*TASH* Tikur Anbessa Specialized Hospital; *Y12HMC* Yekatit 12 Specialized Hospital Medical College; *DRH* Dessie Referral Hospital, *HUCSH* Hawassa University Comprehensive Specialized Hospital; *EOPD* emergency outpatient department; *ICU* intensive care unit; *NICU* neonatal intensive care unit; *BMI* body mass index

### Multivariate analysis of patient demographic characteristics with blood culture finding among patients investigated for sepsis

Possible risk factors for sepsis were identified and their association was assessed using logistic regression. The multivariate analysis showed a statistically significant association of blood culture positivity among age groups < 29 days (*p* = 0.001), ≥ 30 days to ≤ 1 year (*p* = 0.002), > 1 to ≤ 5 years (*p* = 0.000) and > 5 to < 18 years (*p* = 0.001). Medical ward (*p* < 0.019) and pediatric ward (*p* < 0.000) showed statistically significant association for blood culture positivity that yielded growth of pathogens (Table 2). While the univariate analysis indicated statistically significant association with EOPD (*p* < 0.008) with blood culture that yielded pathogens growth, the multivariate analysis did not show statistically significant association. Having underlying disease (*p* < 0.041) was another variable that showed statistically significant association with growth of pathogens. On the other hand, the multivariate analysis did not show any statistically significant association between gender, referral history, previous hospitalization, hospital stay duration, duration of fever, BMI and previous antibiotic treatment with growth of pathogens (Table 2).

**Table 2:** Associations of patient demographic and clinical characteristics with blood culture yield for pathogens

Variables		*P*-value	COR (95%CI)	*P*-value	AOR (95%CI)
Gender	Male	0.801	1.031(0.815–1.303)		
	Female	Constant			
Age category	< 29 days	**0.000**	3.09(2.059–4.099)	**0.001**	1.085(0.363–3.248)
	≥ 30 days to ≤ 1 year	**0.034**	2.905(2.059–4.099)	**0.002**	1.802(0.462–7.020)
	> 1 to ≤ 5 years	**0.008**	1.991(1.302–3.045)	**0.000**	0.570(0.186–1.743)
	> 5 to < 18 years	**0.016**	3.271(2.084–5.134)	**0.001**	0.999(0.311–3.212)
	≥ 18 years	Constant			
Ward	EOPD	**0.008**	3.39(1.384–8.304)	**0.027**	3.597(1.159–11.169)
	ICU	0.156	2.143(0.747–6.149)		
	Medical ward	**0.011**	2.952(1.283–6.792)	**0.004**	4.554(1.609–12.895)
	NICU	0.712	0.872(0.421–1.805)		
	Pediatrics	**0.018**	2.45(1.164–5.155)	**0.002**	7.648(.143–27.287)
	Surgical ward	Constant			
	Outpatient	Constant			
Referral patient	Yes	0.382	1.11(0.879–1.402)		
Previous admission	Yes	0.077	1.308(0.971–1.761)		
Hospital stay duration	1 week	0.124	0.765(0.544–1.076)		
	2 weeks	0.900	1.032(0.633–1.682)		
	3 weeks	0.869	1.037(0.671–1.603)		
	4 weeks and above	Constant			
Underlying diseases	Yes	**0.01**	1.365(1.078–1.728)	**0.012**	0.590(0.391–0.890)
Previous antibiotic treatment before recruitment to the study	Yes	0.785	1.036(0.805–1.332)		
Duration of fever	Up to 3 days	**0.034**	1.478(1.030–2.121)	0.232	1.365(0.819–2.275)
	4–6 days	**0.001**	2.044(1.342–3.114)	0.184	1.447(0.839–2.495)
	7 days and above	Constant			
BMI class	Underweight	0.099	1.899(0.887–4.067)		
	Normal	**0.044**	2.291(1.021–4.067)	**0.045**	2.911(1.022–8.292)
	Overweight	0.438	1.571(0.502–4.919)		
	Obese	Constant			

Bold *p*-value- statistical significant association

*COR* crude odds ratio, *AOR* adjusted odds ratio; *CI* confidence interval, *BMI* body mass index, *EOPD* emergency outpatient department; *ICU* intensive care unit; *NICU* neonatal intensive care unit

Remark: for all yes or no variables, the category “no” considered as constant

### Frequency and distribution of bacterial isolates

**Fig. 1:**
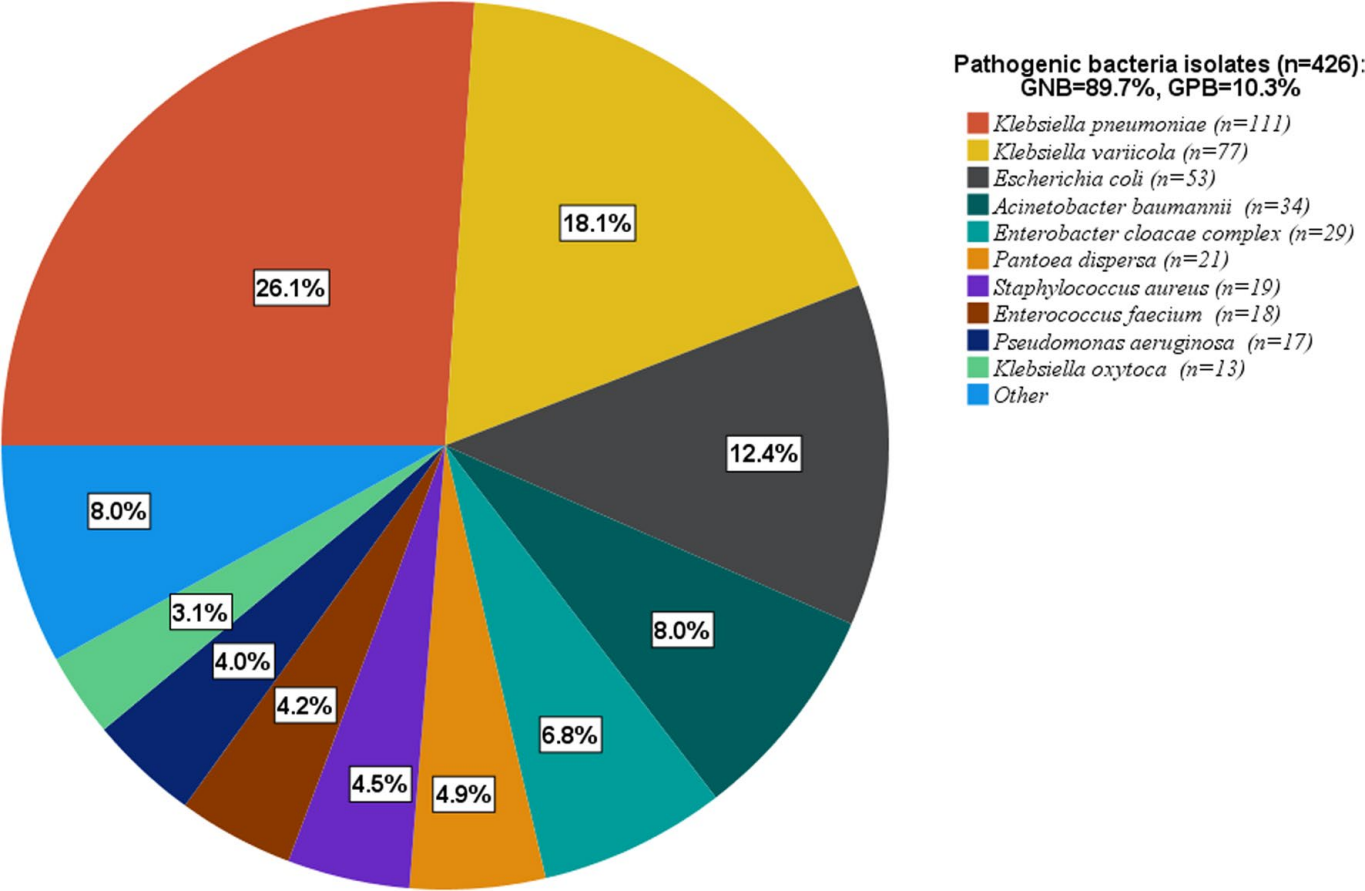
Frequency and distribution of bacteria isolated from patients investigated for sepsis at four different hospitals in Ethiopia. GNB—gram-negative bacteria; GPB—gram-positive bacteria; Other included: *Klebsiella rhinoscleromatis* (n = 3), *Acinetobacter nosocomialis* (n = 2), *Acinetobacter* species (n = 2), *Enterobacter kobei* (n = 2), *Leclercia adecarboxylata* (n = 2), *Raoultella ornithinolytica* (n = 2), *Salmonella* sp (n = 2), *Serratia marcescens* (n = 2), *Stenotrophomonas maltophilia* (n = 2), *Achromobacter xylosoxidans* (n = 1), *Acinetobacter johnsonii* (n = 1), *Acinetobacter lwoffii* (n = 1), *Acinetobacter schindleri* (n = 1), *Acinetobacter ursingii* (n = 1), *Burkholderia cepacia* (n = 1), *Kosakonia cowanii* (n = 1), *Lelliottia amnigena* (n = 1), *Pseudomonas monteillii* (n = 1), *Pseudomonas putida*_Group (n = 1), *Staphylococcus lugdunensis* (n = 4), *Enterococcus faecalis* (n = 1), *Enterococcus italicus* (n = 1), *Enterococcus species* (n = 1), *Enterobacter cloacae* complex included: *Enterobacter cloacae* (n = 22), *Enterobacter xiangfangensis* (n = 3), *Enterobacter bugandensis* (n = 1), *Enterobacter ludwigii* (n = 1)

A total of 426 pathogenic bacteria were isolated from all blood cultures. Gram-negative isolates (89.7%) were the most frequent, while gram-positive isolates accounted for 10.3% (Fig. 1). Double bacterial growth was detected in 41 cultures. *Klebsiella pneumoniae* was most frequent (26.1%), followed by *Ksiella variicola* (18.1%) and *Escherichia coli* (12.4%). Other less frequently detected species were *Acinetobacter baumannii,* 8%, *Enterobacter cloacae,* 5.2%*, Pantoea dispersa,* 4.9%*, Pseudomonas aeruginosa,* 4.0% and *Klebsiella oxytoca,* 3.1%. Pathogenic gram-positive isolates were identified only at TASH and HUCSH and gram-positive isolates detected at Y12HMC and DRH were considered possible contaminants. Diverse species of *Acinetobacter, Enterobacter, Enterococcus, Klebsiella* and *Pseudomonas* were identified. While *K. pneumoniae* was found at all four hospitals, *K. variicola* was mainly detected at DRH (61%) and HUCSH (36.4%), with only 2.6% isolated at TASH and none at Y12HMC. Almost all *P. dispersa* (95.2%) were isolated at DRH and only one *P. dispersa* strain was isolated at TASH, with none detected at Y12HMC or HUCSH. In addition, *Leclercia adecarboxylata*, *Raoultella ornithinolytica*, *Stenotrophomonas maltophilia*, *Achromobacter xylosoxidans, Burkholderia cepacia, Kosakonia cowanii* and *Lelliottia amnigena* were detected as rare bacterial pathogens. Figure 1 shows the frequency of bacterial isolates identified at the four hospitals.

**Fig. 2:**
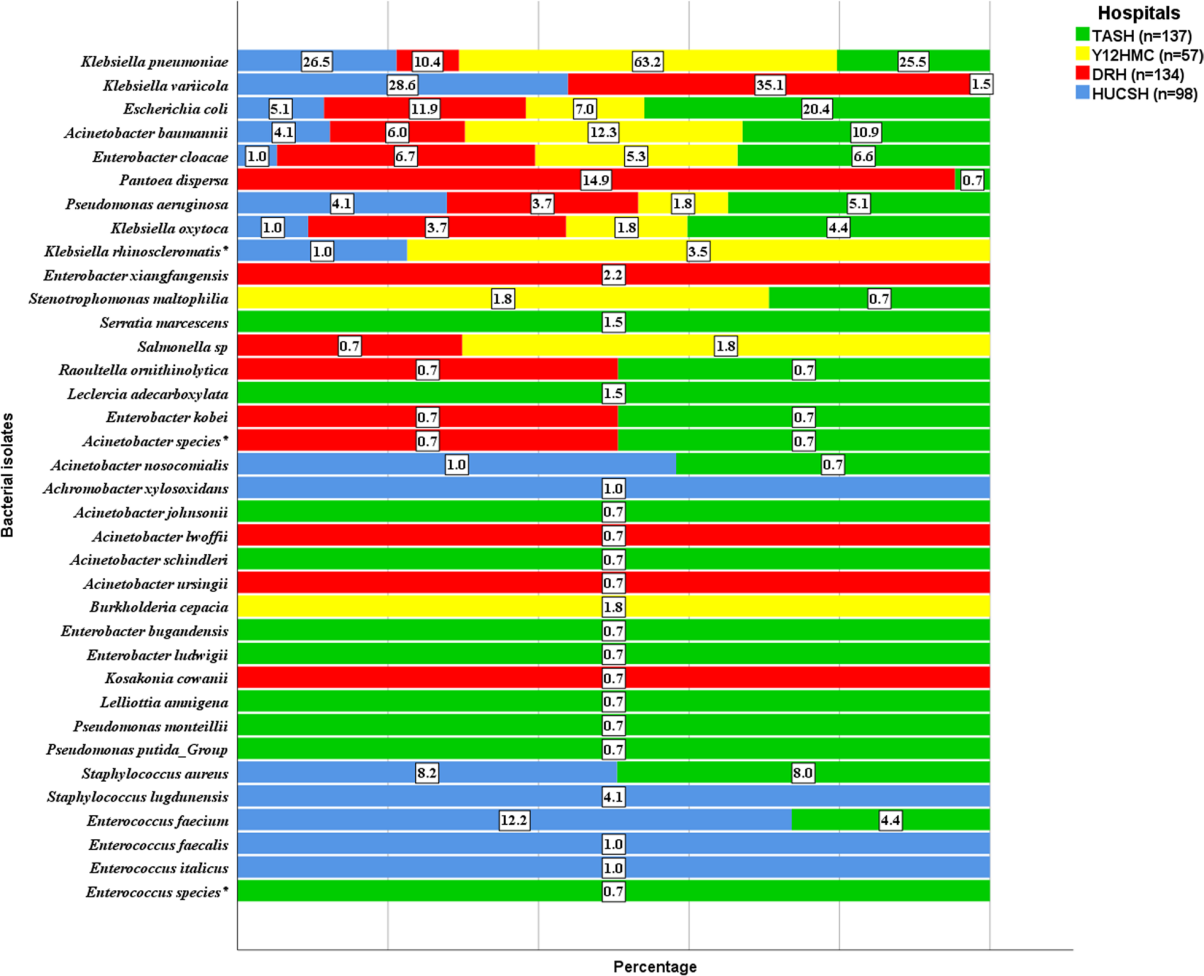
Frequency and distribution of bacterial isolates from the total number of bacteria isolated at each hospital. TASH—Tikur Anbessa Specialized Hospital; Y12HMC—Yekatit 12 Specialized Hospital Medical College; DRH—Dessie Referral Hospital; HUCSH—Hawassa University Comprehensive Specialized Hospital; * reported as identified by phenotypic characterization since MALDI-TOF failed to identify these strains

Among the total bacteria isolated (n = 137) at TASH, *K. pneumoniae, E. coli* and *A. baumannii* were frequent sepsis etiologies, with a proportion of 25.5%, 20.4% and 10.9%, respectively (Fig. 2). Other common isolates were *S. aureus* (8%), *E. cloacae (*6.6%), *P. aeruginosa* (5.1%), *K. oxytoca* (4.4%) and *E. faecium* (4.4%). *S. marcescens* (n = 2) strains were detected at TASH only. *S. maltophilia* (n = 1), *R. ornithinolytica* (n = 1) and *L. amnigena* (n = 1) were other rare sepsis pathogens.

At Y12HMC (n = 57), *K. pneumoniae* and *A. baumannii* were frequently isolated pathogens with a proportion of 63.2% and 12.3%, respectively. A *Salmonella* species (n = 1) was isolated as a rare pathogen, with *B. cepacia* (n = 1) and *S. maltophilia* (n = 1) identified as rare sepsis pathogens.

At DRH, the most frequent pathogenic bacteria were *K. variicola* with a proportion of 35.1%, followed by *P. dispersa* (14.9%), *E. coli* (11.9%) and *K. pneumoniae* (10.4%). Almost all *P. dispersa* were detected at this hospital. *Salmonella* species (n = 1), *R. ornithinolytica* (n = 1) and *K. cowanii* (n = 1) were rare sepsis pathogens at DRH. No pathogenic gram-positive isolates were detected at this hospital and all gram-positive isolates were considered to be possible contaminants.

At HUCSH, *K. variicola* (28.6%) was the most frequent species, followed by *K. pneumoniae* with a proportion of 26.5%. While *Enterobacter* species were commonly isolated in other hospitals, only one *E. cloacae* strain was detected at HUCSH. Most *Enterococcus* species were detected at HUCSH, with *E. faecium* identified frequently at 12.2%. Rarely identified *Enterococcus* species were *E. italicus* and *E. faecalis*. *S. aureus* was another gram-positive coccus identified at this hospital, with a proportion of 8.2%*.* Figure 2 shows the frequency and distribution of bacterial isolates identified at each hospital.

### Antimicrobial resistance pattern of bacterial isolates

**Fig. 3:**
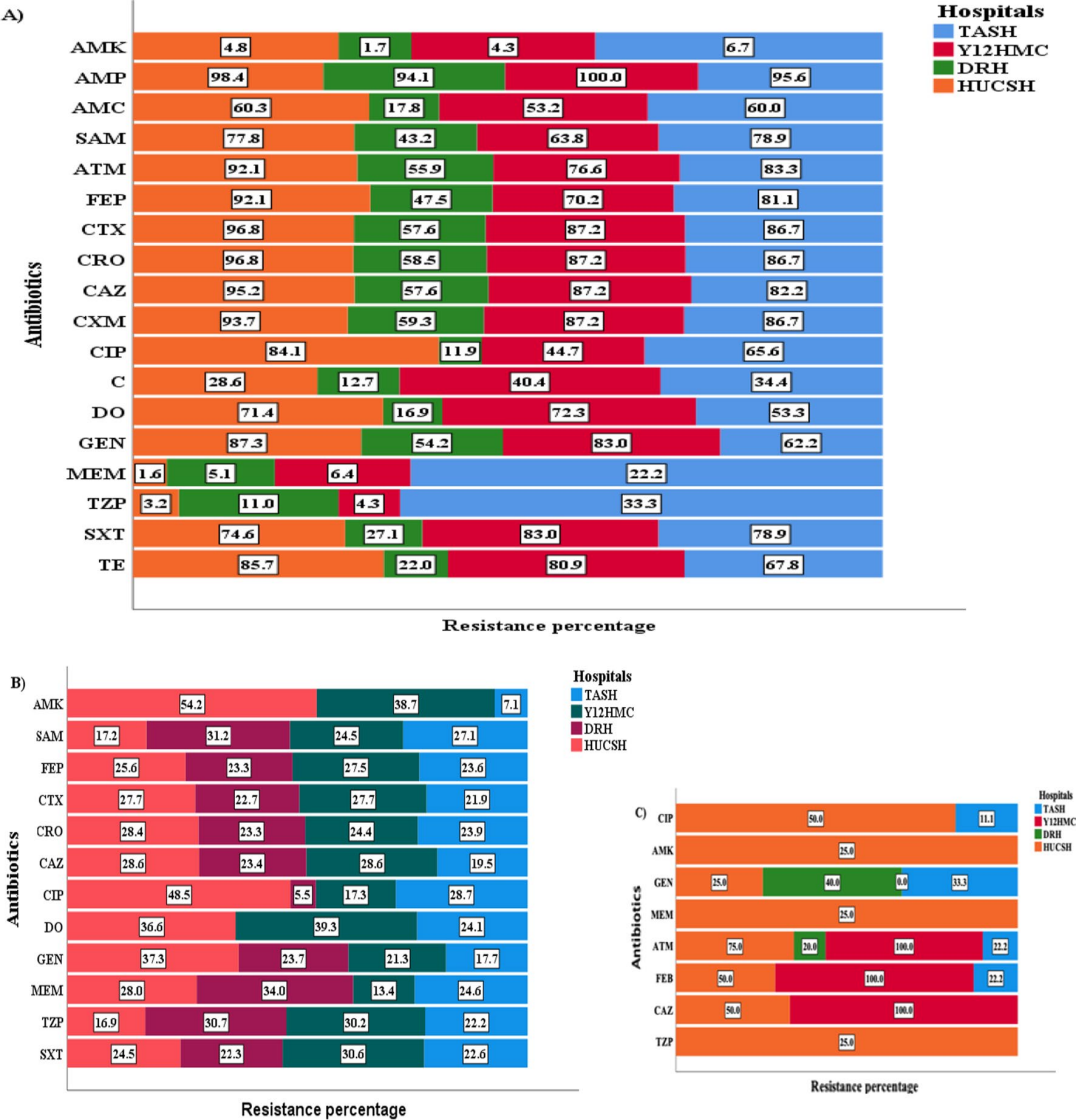
Frequency of antibiotic resistance at the four hospitals **A** *Enterobacteriaceae* **B** *Acinetobacter* species **C** *Pseudomonas* species. Percentage represents number of resistant isolates in relation to total number of isolates at each hospital. AMK—amikacin; AMP—ampicillin; AMC—amoxicillin/clavulanate; SAM—ampicillin-sulbactam; ATM—aztreonam; FEP—cefepime; CTX—cefotaxime; CRO—ceftriaxone; CAZ—ceftazidime; CXM—cefuroxime; CIP—ciprofloxacin; C—chloramphenicol; DO—doxycycline; GEN—gentamicin; MEM—meropenem; TZP—piperacillin/tazobactam; SXT—trimethoprim-sulfamethoxazole; TE—tetracycline; TASH—Tikur Anbessa Specialized Hospital; Y12HMC—Yekatit 12 Specialized Hospital Medical College; DRH—Dessie Referral Hospital, HUCSH—Hawassa University Comprehensive Specialized Hospital

High frequencies of resistance to ampicillin (96.2%), ceftriaxone (78.3%), cefotaxime (78%), cefuroxime (78%), ceftazidime (76.4%), aztreonam (73.9%), cefepime (69.2%), gentamicin (67.3%) and ampicillin-sulbactam (63.2%) were observed among *Enterobacteriaceae* (Table 3). Minimal resistance frequency of *Enterobacteriaceae* was detected to piperacillin/tazobactam (14.8%), meropenem (9.4%) and amikacin (4.1%). The resistance of *Enterobacteriaceae* to meropenem at TASH, Y12HMC, DRH and HUCSH was 22.2%, 6.4%, 5.1% and 1.6%, respectively (Fig. 3A). *Enterobacteriaceae* collected at DRH showed a resistance of less than 60% to tested antibiotics except ampicillin, to which resistance was 94.1%. Many *Enterobacteriaceae* showed intermediate patterns of susceptibility to most antibiotics. Table 3 shows the resistance and intermediate patterns of antibiotics tested against *Enterobacteriaceae*.

**Table 3:** Antimicrobial resistance pattern of *Enterobacteriaceae, Acinetobacter* and *Pseudomonas* species isolated from patients investigated for sepsis

*Gram negative isolates *	AMK		AMP		AMC		SAM		ATM		FEP		CTX		CRO		CAZ	
	I %	R %	I %	R %	I %	R %	I %	R %	I %	R %	I %	R %	I %	R %	I %	R %	I %	R %
*K. pneumoniae* (n = 111)	15.3	7.2	0.9	99.1	24.3	55.9	13.5	73	4.5	93.7	9.0	83.8	0	96.4	0	96.4	0.9	95.5
*K. variicola* (n = 77)	18.2	0	1.3	98.7	27.3	29.9	11.7	53.2	3.9	63.6	3.9	62.3	0	67.5	0	67.5	0	67.5
*E. coli* (n = 53)	7.5	3.8	5.7	90.6	30.2	34	9.4	62.3	3.8	60.4	0	60.4	1.9	64.2	1.9	64.2	1.9	62.3
*E. cloacae* complex (n = 29)	20.7	3.4	3.4	93.1	6.9	79.3	6.9	65.5	0	62.1	6.9	51.7	3.4	58.6	6.9	58.6	0	58.6
*P. dispersa* (n = 21)	4.8	4.8	0	95.2	61.9	28.6	4.8	81.0	0	100	4.8	85.7	0	95.2	0	100	0	95.2
*K. oxytoca* (n = 13)	7.7	0	0	100	53.8	23.1	38.5	46.2	15.4	53.8	7.7	69.2	7.7	76.9	7.7	76.9	15.4	61.5
Rare *Enterobacteriaceae* isolates (n = 14)	0	7.1	7.1	85.7	35.7	21.4	21.4	28.6	7.1	28.6	14.3	35.7	7.1	57.1	14.3	57.1	14.3	50.0
Total *Enterobacteriaceae *(n = 318)	13.5	4.1	2.2	96.2	28.6	43.4	12.6	63.2	4.1	73.9	6.0	69.2	1.3	78.0	1.9	78.3	1.9	76.4
*Acinetobacter* species																		
*Acinetobacter baumannii* (n = 34)	8.8	14.7	–	–	–	–	2.9	70.6	–	–	2.9	82.4	5.9	91.2	11.8	88.2	5.9	85.3
Other *Acinetobacter* species (n = 8)	0	0	–	–	–	–	0	25	–	–	12.5	50	37.5	62.5	25	75	12.5	62.5
Total *Acinetobacter species* (n = 42)	7.1	11.9	–	–	–	–	2.4	61.9	–	–	4.8	76.2	11.2	85.7	14.3	85.7	7.1	81
*Pseudomonas* species																		
*P. aeruginosa* (n = 17)	5.9	5.9	–	–	–	–	–	–	29.4	35.3	0	29.4	–	–	–	–	17.6	17.6
Other *Pseudomonas* species (n = 2)	0	0	–	–	–	–	–	–	50	50	100	0	–	–	–	–	0	0
Total *Pseudomonas* species (n = 19)	5.3	5.3	–	–	–	–	–	–	31.6	36.8	10.5	26.3	–	–	–	–	15.8	15.8

*Gram negative isolates *	CXM		CIP		C		DO		GEN		MEM		TZP		SXT		TE	
	I %	R %	I %	R %	I %	R %	I %	R %	I %	R %	I %	R %	I %	R %	I %	R %	I %	R %
*K. pneumoniae* (n = 111)	1.8	94.6	2.7	59.5	1.8	43.2	4.5	65.8	1.8	82.0	0	17.1	9.0	18.0	0.9	85.6	1.8	70.3
*K. variicola* (n = 77)	0	68.8	0	37.7	1.3	5.2	5.2	32.5	1.3	67.5	0	1.3	7.8	3.9	0	36.4	0	41.6
*E. coli* (n = 53)	0	66	0	50.9	0	24.5	7.5	58.5	5.7	49.1	0	1.9	1.9	20.8	0	66	0	73.6
*E. cloacae* complex (n = 29)	3.4	58.6	3.4	44.8	3.4	37.9	13.8	37.9	3.4	44.8	3.4	24.1	3.4	31.0	0	55.2	6.9	55.2
*P. dispersa* (n = 21)	0	95.2	0	9.5	0	0	0	4.8	0	90.5	0	9.5	0	14.3	9.5	4.8	0	4.8
*K. oxytoca* (n = 13)	0	76.9	0	46.2	0	30.8	15.4	23.1	0	61.5	0	0	0	0	0	61.5	0	53.8
Rare *Enterobacteriaceae* isolates (n = 14)	0	57.1	14.3	28.6	0	21.4	7.1	21.4	7.1	35.7	0	0	0	7.1	0	42.9	0	42.9
Total *Enterobacteriaceae* (n = 318)	0.9	78.0	1.9	46.2	1.3	26.1	6.3	46.2	2.5	67.3	0.3	9.4	5.7	14.8	0.9	59.4	1.3	56.3
*Acinetobacter* species																		
*Acinetobacter baumannii* (n = 34)	–	–	0	41.2	–	–	2.9	26.5	0	64.7	0	58.8	8.8	67.6	2.9	82.4	–	–
Other *Acinetobacter* species (n = 8)	–	–	12.5	25	–	–	0	12.5	12.5	37.5	0	37.5	0	25	0	62.5	–	–
Total *Acinetobacter* species (n = 42)			2.4	38.1			2.4	23.8	2.4	59.5	2.4	54.8	7.1	59.5	2.4	78.6	–	–
*Pseudomonas* species																		
*P. aeruginosa* (n = 17)	–	–	11.8	17.6	–	–	–	–	5.9	35.3	0	5.9	0	5.9	–	–	–	–
Other *Pseudomonas* species (n = 2)	–	–	0	0	–	–	–	–	0	0	0	0	0	0	–	–	–	–
Total *Pseudomonas* species (n = 19)	–	–	10.5	15.8	–	–	–	–	5.3	31.6	0	5.3	5.3	15.8	–	–	–	–

*R* resistance; *I* intermediate; *AMK* amikacin; *AMP* ampicillin; *AMC* amoxicillin/clavulanate; *SAM* ampicillin-sulbactam; *ATM* aztreonam; *FEP* cefepime; *CTX* cefotaxime; *CRO* ceftriaxone; *CAZ* ceftazidime; *CXM* cefuroxime; *CIP* ciprofloxacin; *C* chloramphenicol; *DO* doxycycline; *GEN* gentamicin; *MEM* meropenem; *TZP* piperacillin/tazobactam; *SXT* trimethoprim-sulfamethoxazole; *TE* tetracycline

*K. pneumoniae* showed highest resistance to ampicillin (99.1%), cefotaxime (96.4%), ceftriaxone (96.4%), ceftazidime (95.5%), cefuroxime (94.6%), aztreonam (93.7%), cefepime (83.8%), trimethoprim-sulfamethoxazole (SXT) (85.6%) and gentamicin (82%). Resistance to meropenem was 17.1% for *K. pneumoniae,* while *K. variicola* showed comparatively lower frequencies of resistance. Among 77 *K. variicola* isolates, only one showed resistance to meropenem. *E. coli* showed > 60% resistance to ampicillin, cefuroxime, cefotaxime, ceftriaxone, ceftazidime, ampicillin-sulbactam, aztreonam, cefepime, SXT and tetracycline.

In the non-fermenter group, *A. baumannii* showed highest resistance to cefotaxime (91.2%), ceftazidime (85.3%), cefepime (82.4%) and SXT (82.4%). *A. baumannii* also showed high resistance to ampicillin-sulbactam (70.6%), piperacillin-tazobactam (67.6%), gentamicin (64.7%) and meropenem (58.8%) (Table 3). The resistance frequency of *Acinetobacter* species to meropenem at DRH, HUCSH, TASH and Y12HMC was 34.0%, 28%, 24.6% and 13.4%, respectively (Fig. 3B). On the other hand, *P. aeruginosa* showed low resistance to tested antibiotics (Table 3, Fig. 3C). Figure 3 shows the total antibiotic resistance pattern of *Enterobacteriaceae*, *Acinetobacter* species and *Pseudomonas* species, by hospital.

*S. aureus* showed a resistance of 94.7%, 78.9%, 63.2% and 57.9% to penicillin, erythromycin, tetracycline and SXT, respectively. Cefoxitin, which is a surrogate marker of methicillin, showed inefficacy of 47.4% against *S. aureus*. *E. faecium* showed 100% resistance to penicillin and ampicillin. It also showed a resistance of 61.1% to vancomycin and 50% to erythromycin. Table 4 shows the AMR pattern of gram-positive bacteria.

**Table 4:** Antimicrobial resistance pattern of gram-positive bacteria isolated from patients investigated for sepsis

Gram-positive isolates	Penicillin	Ampicillin	Vancomycin	Chloramphenicol		Erythromycin		Ciprofloxacin		Gentamicin		Trimethoprim sulfamethoxazole		Tetracycline		Cefoxitin	Clindamycin	
	R %	R %	R %	I %	R %	I %	R %	I %	R %	I %	R %	I %	R %	I %	R %	R %	I %	R %
*S. aureus* (n = 19)	94.7	NA	NA	21	36.8	0	78.9	21	26.3	15.6	31.6	15.6	57.9	10.5	63.2	47.4	10.5	47.4
*S. lugdunensis* (n = 4*)*	100	NA	NA	0	25	25	75	0	0	0	75	25	750	25	750	0	25	750
Total (n = 23)	95.7	NA	NA	17.4	34.8	4.3	78.3	17.4	21.7	13	39.1	17.4	56.5	13	60.9	39.1	13	47.8
*E. faecium* (n = 18)	100	100	61.1	16.7	27.8	33.3	50	NA	NA	NA	NA	NA	NA	NA	NA	NA	NA	NA
*Other Enterococcus species* (n = 3)	100	33.3	100	0	100	100	0	NA	NA	NA	NA	NA	NA	NA	NA	NA	NA	NA
Total (n = 21)	100	90.5	66.7	14.3	38.1	42.9	42.9	NA	NA	NA	NA	NA	NA	NA	NA	NA	NA	NA

*NA* non applicable; *R* resistance; *I* intermediate

### Multidrug resistance level

**Fig. 4:**
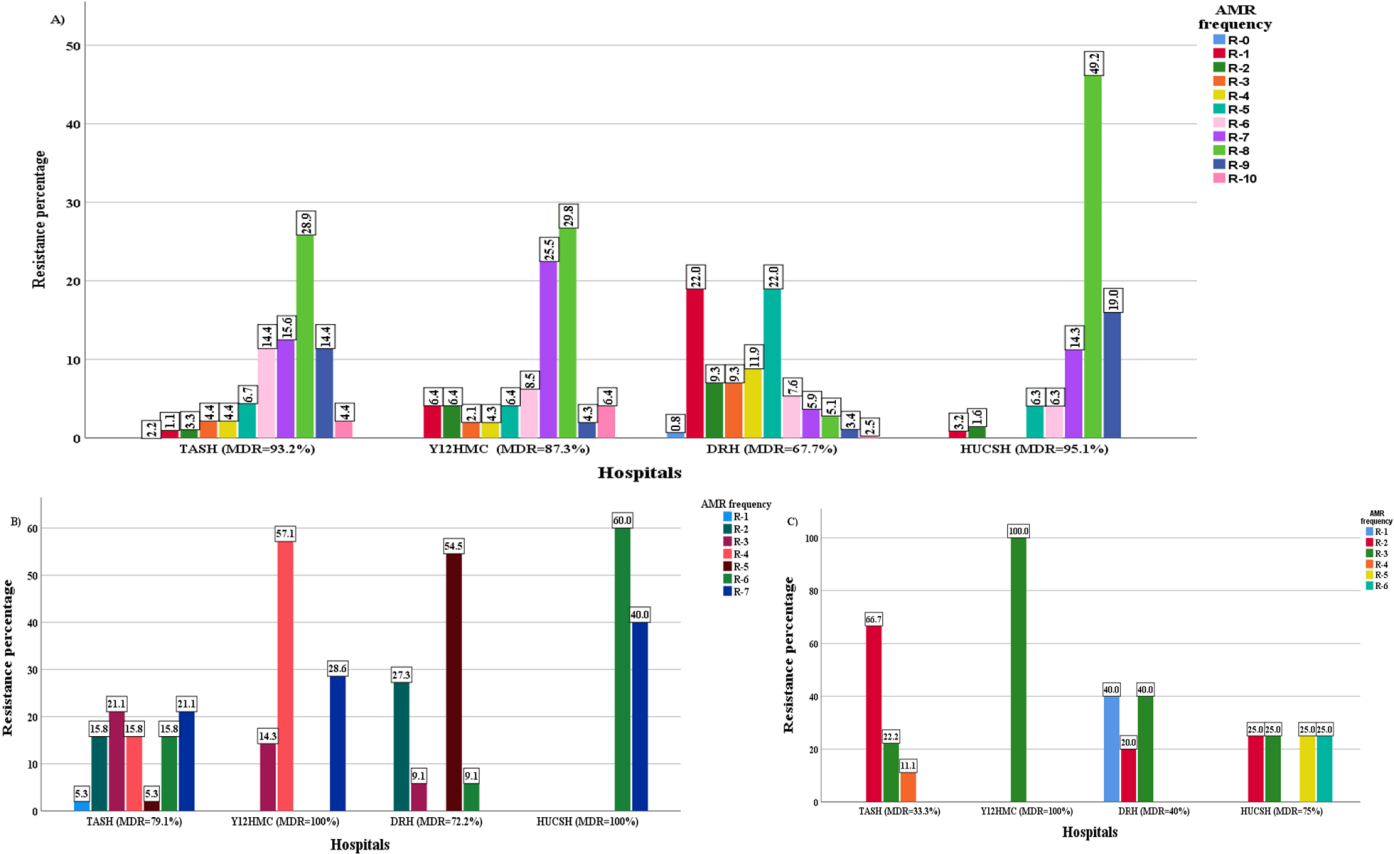
Frequency of multidrug resistance at four hospitals. **A** *Enterobacteriaceae* **B** *Acinetobacter* species **C** *Pseudomonas* species. Percentages represent number of resistant isolates in relation to total number of isolates at each hospital. TASH—Tikur Anbessa Specialized Hospital; Y12HMC—Yekatit 12 Specialized Hospital Medical College; DRH—Dessie Referral Hospital, HUCSH—Hawassa University Comprehensive Specialized Hospital; MDR—multidrug resistance

*Enterobacteriaceae* showed an overall MDR frequency of 83.2% (Table 5) which was a very high MDR level. *Enterobacteriaceae* that showed MDR to eight (R-8), nine (R-9) and ten (R-10) antibiotics from different groups had a frequency of 24.2%, 9.7% and 3.1%, respectively. Only 0.9% *Enterobacteriaceae* showed zero resistance (R-0) to all antibiotic classes tested, while 10.1% *Enterobacteriaceae* showed resistance to one antibiotic (R-1) class. For *Enterobacteriaceae*, the MDR frequency at HUCSH, TASH, Y12HMC and DRH was 95.1%, 93.2%, 87.3%, and 67.7%, respectively (Fig. 4A). *K. pneumoniae, E. coli, K. oxytoca, P. dispersa, E. cloacae and K. variicola* showed an overall MDR frequency of 95.5%, 84.9%, 84.6%, 81%, 72.2% and 71.4%, respectively. The overall MDR frequency of *A. baumannii* was 91.2%. The MDR frequency for *Acinetobacter species* was 100% at HUCSH and Y12HMC, while it was 79.1% at TASH and 72.2% at DRH (Fig. 4B). On the other hand, MDR frequency for *Pseudomonas* species was 100%, 75%, 33.3% and 40% at Y12HMC, HUCSH, TASH and DRH, respectively (Fig. 4C). An overall MDR frequency of 58.8% was detected among *P. aeruginosa.*

**Table 5:** Multidrug resistance frequency of *Enterobacteriaceae, Acinetobacter* and *Pseudomonas* species

Gram-negative isolates	Resistance frequency												
		R-0%	R-1%	R-2%	R-3%	R-4%	R-5%	R-6%	R-7%	R-8%	R-9%	R-10%	MDR %
*Enterobacteriaceae*	*K. pneumoniae* (n = 111)	0	0.9	3.6	0.9	0.9	8.1	15.3	15.3	34.2	12.6	8.1	95.4
	*K. variicola* (n = 77)	0	22.1	6.5	3.9	14.3	9.1	2.6	7.8	28.6	5.2	0	72.2
	*E. coli* (n = 53)	3.8	7.5	3.8	7.5	11.3	17.0	1.9	22.6	13.2	11.3	0	84.8
	*E. cloacae* complex (n = 29)	3.4	10.3	13.8	10.3	0	6.9	10.3	3.4	24.1	13.8	3.4	72.7
	*P. dispersa* (n = 21)	0	9.5	9.5	14.3	4.8	42.9	14.3	0	0	4.8	0	81
	*K. oxytoca* (n = 13)	0	15.4	0	7.7	0	7.7	15.4	30.8	15.4	7.7	0	84.6
	Rare *Enterobacteriaceae* isolates (n = 14)	0	21.4	7.1	7.1	7.1	14.3	14.3	14.3	7.1	7.1	0	71.3
	Total (n = 318)	0.9	10.1	5.7	5.0	6.3	12.3	9.4	13.2	24.2	9.7	3.1	83.2
*Acinetobacter* species	*A. baumannii* (n = 34*)*	0	2.9	5.9	14.7	20.6	20.6	14.7	20.6	–	–	–	91.2
	Other *Acinetobacter* species *(n* = *8)*	0	0	50.0	12.5	0	0	25	12.5	–	–	–	50.0
	Total (n = 42)	0	2.4	14.3	14.3	16.7	16.7	16.7	19.0	–	–	–	83.4
*Pseudomonas* species	*P. aeruginosa* (n = 17*)*	0	11.8	29.4	29.4	23.5	0	5.9	–	–	–	–	58.8
	Other *Pseudomonas* species (n = 2)	0	0	100	0	0	0	0	–	–	–	–	0
	Total (n = 19)	0	10.5	42.1%	31.6	5.3	5.3	5.3	–	–	–	–	47.5

R-0, R-1, R-2, …, R-10 stand for resistance to 0, 1, 2, …., 10 antibiotics from different classes; MDR stands for multidrug resistance to three or more antibiotics from different classes,—non applicable

## Discussion

In the current study, the overall pathogenic bacterial growth among patients investigated for sepsis was 27.2%, with only 0.3% due to yeast cells. In total, 13.1% possible contaminants yielded growth, of which some could be real pathogens, especially for neonates, patients who had underlying diseases and immunocompromised patients [8]. There was substantial variability in the frequency of positive blood cultures among patients investigated for sepsis between hospitals: 38.2% in northern Ethiopia, 29.1% in southern Ethiopia and 24.6% and 18.5% at the two hospitals in central Ethiopia. Explanations could be institutional infection prevention strategies and their implementation, professional capacity to identify sepsis cases early, awareness of sepsis occurrence rate and diagnosis within institutions, and availability and utilization of laboratory facilities [2, 17, 26].

The most frequent sepsis etiologies were *K. pneumoniae* (26.1%), *K. variicola* (18.1%) and *E. coli* 12.4%. *Klebsiella* species have been found to cause substantial morbidity in sub-Saharan Africa [12]. Our finding was in line with a study from India that reported gram-negative bacteria as the primary sepsis etiologies, with 23% of sepsis due to *K. pneumoniae* alone [27]. While *E. coli* was the third most frequent of all blood culture isolates, most were isolated at TASH and DRH, and rarely at the other hospitals. Similar studies have shown that *E. coli* is a commonly detected pathogen in sepsis patients [16, 28]. Other common gram-negative isolates were *A. baumannii* 8%*, E. cloacae 5.2*%*, P. dispersa* 4.9% and *P. aeruginosa 4%*. *S. aureus* and *E. faecium* were commonly detected gram-positive isolates, mirroring results of other studies elsewhere though they were isolated at TASH and HUCSH only [17, 28, 29].

Notable disparities on the type and frequency of bacteria isolated as sepsis etiologies were seen between hospitals. While *K. pneumoniae* was the primary sepsis etiology at TASH and Y12HMC, *K. variicola* was the primary causative agent of sepsis at DRH and HUCSH. Almost all *K. variicola* were detected at these two hospitals, located in the north and south of the country. This species is currently recognized as an emerging pathogen that can cause severe infections in humans, and can also colonize plants, insects and animals [30]. A study from Stockholm showed *K. variicola* as a common blood stream pathogen, though it was inaccurately identified as *K. pneumoniae* initially using classical species identification methods [31]. Almost all *P. dispersa* 95.2% were isolated in the northern part of the country, at DRH. This was the second most frequent isolate in blood cultures from this site. It was believed that *P. dispersa* lives in plants, soil and water and rarely caused human infections until a first neonatal sepsis was reported from India [32] and later adult sepsis in Japan [33]. Another case report from India showed that *P. dispersa* was detected in a septic patient admitted to ICU; this was reported as the next emerging ICU scare [34]. In our study, most *P. dispersa* were isolated at the neonatal ICU while two strains were isolated from adult patients admitted to the emergency outpatient department at DRH. Only one strain was isolated at TASH (central Ethiopia); this was in an adult patient hospitalized in a medical ward.

Uncommon sepsis etiologies identified were *A*. *xylosoxidans, B. cepacia, L. amnigena, K. cowanii, S. maltophilia* and *R. ornithinolytica*. Such emerging pathogens in patients investigated for sepsis could create challenges in the future [35–37]. This is the first time these species, together with *K. variicola* and *P. dispersa*, were reported in patients investigated for sepsis in Ethiopia. Isolation of such emerging sepsis etiologies highlights the need for institutional-based diagnostic and intervention strategies.

*Enterobacteriaceae* showed an MDR frequency of 83.3% and a majority of them were frequently resistant to ampicillin, ampicillin-sulbactam, aztreonam, ceftriaxone, cefotaxime, cefuroxime, ceftazidime, cefepime and gentamicin. Carbapenem resistance among *Enterobacteriaceae* was 9.4%. Available effective treatment options for *Enterobacteriaceae* were limited to piperacillin-tazobactam, amikacin and meropenem. *Enterobacteriaceae* isolated at DRH showed low resistance to tested antibiotics compared with *Enterobacteriaceae* isolated at other hospitals. The occurrence of higher antibiotic resistance among *Enterobacteriaceae* was in line with studies from other countries [9, 10, 12]. *K. pneumoniae,* the most frequent isolate, showed the highest resistance (> 80%) to ampicillin, cefotaxime, ceftriaxone, ceftazidime, cefuroxime, aztreonam, cefepime, SXT and gentamicin. In our study, an alarming level of carbapenem-resistant *K. pneumoniae* (17.1%) was detected, though it was lower than in another study [13]. *A. baumannii* showed high resistance (> 80%) to cefotaxime, ceftazidime, cefepime and SXT, with an overall MDR frequency of 91.2%. This high MDR frequency of *A. baumannii* was in line with a study from India [13. *A. baumannii* also showed a remarkably high resistance to meropenem (58.8%). Our findings had similarities with studies from Asia [11, 13]. The occurrence of meropenem resistance among infrequently detected *A. nosocomialis* could be a sign of spreading of carbapenem resistance between *Acinetobacter* species.

The strengths of this study include the selection of hospitals in multiple parts of the country, enrollment of all age groups, a reasonably large sample size and re-characterizing bacteria using an advanced bacterial identification method. On the other hand, the following three limitations should be considered. First, additional sepsis cases might be missed when data collectors were unavailable during blood sample collection. Second, it was impossible to use two or three blood cultures, which could have increased the growth yield and enabled categorization of coagulase negative staphylococci as true pathogens or contaminants. Third, anaerobic incubation of blood culture that could enable the identification of strict anaerobes was not applied.

## Conclusion

In this multicenter study, frequent and diverse gram-negative sepsis etiologies were identified, with substantial variation in primary etiologies between hospitals in different parts of the country. Gram-negative isolates were the primary causative agents of sepsis at all hospitals. Isolation of emerging bacterial strains in all sites showed the growing epidemiology and diversity of sepsis etiologies. High antimicrobial resistance was detected with varying frequency between hospitals. These findings could be taken as a call for strategies to control sepsis occurrence and curb the spread of multidrug resistance. Strategies to control antimicrobial resistance should include context-specific measures. High-quality bacteriological services capable of monitoring emerging drug-resistant bacterial agents of sepsis are essential for antimicrobial stewardship programs to be effective.

## Data Availability

The datasets used and/or analysed during the current study are available from the corresponding author on reasonable request.
